# 

**Published:** 1904-01-23

**Authors:** 


### Publications.

Just Published. 8vo. with Photographic reproductions, 3/- net.

**THE PHYSIOGNOMY OF MENTAL DISEASES AND DEGENERACY.** By JAMES SHAW M.D., formerly Med. Supt. and Co-Licensee Haydock Lodge Asylum. Reprinted, with alterations and additions, from various articles in the "Medical Annual."

Just Published. Crown 8vo 1/6 net.

**A POCKET BOOK OF CLINICAL METHODS.** By CHAS. H. MELLAND, M.D.Lond., M.R.C.P., Phys. Ancoats Hosp.

THIRD EDITION. 12th Thousand. Small 8vo. Illustrated with 200 Original Drawings, 2/6. Lantern Slides of the Illustrations, 1/- and 1/6 each.

**"FIRST AID" TO THE NJURED AND SICK:** An Advanced Ambulance Handbook. By F. J. WARWICK, B.A., M.B., and A. C. TUNSTALL, M.D., F.R.C.S.

THIRD EDITION. 2/- each, or 27/6 the Set of 16 Sheets, or mounted on linen, 45/- With nickel head.

**(Adopted by the War Office, Admiralty, and London School Board.)**

**LARGE SHEET "FIRST AID" DIAGRAMS:** Being Enlargements of the Illustrations in the above work on sheets 26 in. by 40 in., suitable for lectures and classes.

### MEDICAL ACCOUNTS.

Now is the time to arrange for this troublesome business, and

**WRIGHT'S THREE-BOOK SYSTEM,**
**WRIGHT'S SINGLE-BOOK SYSTEM,**
(11th Edition)
**WRIGHT'S OBSTETRIC RECORDS,**
**WRIGHT'S VISITING LISTS, 1904,**
**WRIGHT'S CHARTS of all kinds,**
(Nearly 400,000 of which have been sold)
**WRIGHT'S CHART HOLDERS,**
(In use all over the world)
**WRIGHT'S CERTIFICATE BOOKS,**
**PROFESSIONAL ACCOUNT FORMS,**
**LETTER HEADINGS, and**
**DISPENSING LABELS.**

Are most of them better than the ordinary run of these things, and their use saves time and trouble.

PLEASE SEND FOR FREE SAMPLES.

**Bristol: JOHN WRIGHT & CO.** **London: SIMPKIN & CO., Ltd.**

---

FOURTH EDITION. Plates contain 93 Coloured and Stereoscopic Figures, 209 Illustrations in the Text. 17/6 net, including Stereoscope.

By P. WATSON WILLIAMS, M.D.(Lond.),

Physician in Charge of Throat Department, Bristol Royal Infirmary, &c., &c.

### DISEASES OF THE NOSE, PHARYNX, AND LARYNX.

WITH A SECTION ON THE EXAMINATION OF

**THE EAR.**

**Press Notices.**—"We know of no better practical guide"—*Practitioner.* "Certain to receive the recommendation of all teachers"—*Brit. Med. Journ.* "We can strongly recommend the book."—*Lancet.* "Excellent.... The features of the work—completeness, clear description, and practical utility—are absolutel maintained in the new edition.—(Transn.)." *Internat. Centr. f. Laryngologie.*

Bristol: J. WRIGHT & CO. London: SIMPKIN & CO.

---

Second Edition. Crown 8vo., price 3/6 net.
With original "Plea—1900—for the State Registration of Nurses."

### MOCK NURSES OF THE LATEST FASHION.

By FREDERICK J. GANT, F.R.C.S.,

Consulting Surgeon to the Royal Free Hospital.

BAILLIÈRE, TINDALL & COX,
Henrietta Street, Covent Garden, London.

---

A LIST of the NEW PUBLICATIONS of the SCIENTIFIC PRESS, LIMITED, will be sent post free to any member of the Medical Profession on receipt of post card.

28 & 29 SOUTHAMPTON STREET STRAND, LONDON, W.C.

---

Crown 8vo. Illustrated. Price **2s. 6d.** net.

**ANÆSTHETICS.** By J. BLUMFELD, M.D.Cantab., Senior Anæsthetist to St. George's Hospital; Anæsthetist to the Grosvenor Hospital for Women and Children, London, and to the National Dental and Metropolitan Hospitals.

*Lancet.*—"....In writing this handbook the author has been quite successful." *Australasian Medical Gazette.*—"We can strongly commend this book." *Medical Chronicle.*—"The book is one which we can heartily recommend to senior students and practitioners."

London: BAILLIÈRE, TINDALL & COX, 8 Henrietta St., Covent Garden.

---

### Hoblyn's Dictionary of TERMS USED IN MEDICINE and the Collateral Sciences.

13th Edition, Revised by J. A. P. PRICE. **10/6.**

"It has a rare merit of supplying in almost every case what you have a right to expect in consulting it."—*Glasgow Medical Journal.*

"As a handy reference volume for the physician, surgeon and pharmacist it will prove invaluable."—*Pharmaceutical Journal.*

---

### THE APOTHECARIES' HALL MANUAL FOR

**Students preparing for the Dispenser's Certificate.**
**By M. THOMSON, 2/- net.**

WHITTAKER & CO., White Hart St., Paternoster Sq., London, E.C.

### NOTICE TO CORRESPONDENTS.

All MSS., Letters, Books for Review, and other matters intended for the Editor should be addressed The Editor, "The Hospital," The Hospital Buildings, 28 & 29 Southampton Street, Strand, W.C.

The Editor cannot undertake to return rejected MS., even when accompanied by stamped directed envelope.

### Notice to Advertisers and Subscribers.

All Advertisements, Orders for Copies of The Hospital, and Business Communications should be addressed to **THE MANAGER (Not to the Editor),** The Hospital, 28 & 29 Southampton Street, Strand, London, W.C.

The Cost of Subscription, post free, is as follows:—Per week, 3½d.; per quarter, 3s. 9d.; per half-year, 7s. 6d.; per annum, 15s. Foreign and Colonial:—Per annum, 19s. 6d., payable, in all cases, in advance.

**NOTICE.—Vol. XXXIV. of "The Hospital" (April 1903 to September 1903), in handsome cloth binding, is now on sale at the office of this Journal, price 10s. 6d. Cases for binding the Numbers contained in this Volume 2s. Index to Vol. XXXIV., 2½d., post free.**

**The Monthly part for January is now ready. Price 1s., by post, 1s. 3d.**

### BOOKS RECEIVED.

H. K. LEWIS.

"Movable or Dropped Kidney." By C. W. Suckling, M.D.

W. E. SMITH.

"Mechanism of the Paroxysmal Neuroses." By F. Hare, M.D.

BAILLIÈRE, TINDALL AND COX.

"Aids to Surgery." By J. Cunning.

WALTER SCOTT PUBLISHING CO.

"Proposed Sterilisation of certain Mental and Physical Degenerates." By Robert R. Rentol.

### Important Works of Reference on Hospitals.

By Sir HENRY BURDETT, K.C.B.

### HOSPITALS AND ASYLUMS OF THE WORLD:

Their Origin, History, Construction, Administration, Management and Legislation; with Plans of the chief Medical Institutions, accurately drawn to a uniform scale.

In Four Volumes, and a Separate Portfolio containing some Hundreds of Plans. Royal 8vo.

| | | |
|---|---|---|
| Top Gilt, cloth extra, bevelled. Price of the Book, complete, ... | £12 12s. 0d. | Strictly net. |
| Vols. I. and II. (only)—Asylums and Asylum Construction ... ... ... ... | £6 15s. 0d. | |
| Vols. III. and IV.—Hospitals and Hospital Construction—with Portfolio of Plans... | £9 0s. 0d. | |
| The Portfolio of Plans (20 ins. by 14 ins.), separately ... ... ... ... ... | £4 14s. 6d. | |

**Important.**—This monumental work can now be obtained on "The Times" plan of Twelve monthly payments of One Guinea each. Prospectus and Order Form post free on application. Only a few copies of the work remain unsold, and this offer will be withdrawn directly these are disposed of.

### COTTAGE HOSPITALS: General, Fever & Convalescent.

Their Progress, Management and Work throughout the world.

Third Edition, profusely Illustrated, with nearly 50 Plans, Diagrams, etc. Crown 8vo. cloth gilt, **10/6** post free.

"A complete manual of all that relates to the founding, the cost and the management of cottage hospitals . . . . No more complete manual could be required."—*St. James's Gazette.*

### BURDETT'S HOSPITALS AND CHARITIES.

The Year Book of Philanthropy and Hospital Annual.

Published Annually. Crown 8vo. over 1,000 pp., cloth gilt, **5/-** net; **5/5** post free.

This work is the recognised Standard Authority on all points relating to Charities, whether British American, or Colonial, and may be regarded as "The Hospital Whitaker."

### THE UNIFORM SYSTEM OF ACCOUNTS,

### For Hospitals and all Classes of Public Institutions.

With Special Forms of Account and Specimen Rulings for a Complete Set of Books.

New Edition. Royal 8vo. cloth. **4/-** net; **4/4** post free.

### ACCOUNT BOOKS FOR INSTITUTIONS.

Designed in accordance with the Uniform System of Accounts for Hospitals and all Classes of Public Institutions.

*Full Description of Books and Prices Post Free on Application.*

### HOSPITAL EXPENDITURE: THE COMMISSARIAT.

For Hospital Matrons and Managers. Crown 8vo. cloth, **2/6** post free.

"This should prove a valuable handbook to those upon whom rests the responsibility of hospital finance, and should be of interest to the public as giving a fair idea of what this department should and does cost."—*Manchester Courier.*

*Of all Booksellers, or of*

THE SCIENTIFIC PRESS, LTD., 28 & 29 Southampton Street, Strand, London, W.C.

### The Student of Medicine.

### APPOINTMENTS.

**W. G. Armstrong**, M.B., Ch.M.Syd., Lecturer on Public Health at the University of Sydney. **F. J. Barker**, M.B., C.M., Clinical Assistant to the Chelsea Hospital for Women. **Harold Barwell**, M.B.Lond., F.R.C.S.Eng., Assistant Surgeon to the Metropolitan Ear, Nose, and Throat Hospital, Grafton Street, Fitzroy Square. **N. Bennett**, M.D.Durh., District Medical Officer of the Newcastle-upon-Tyne Union. **W. J. Davies**, M.R.C.S, L.R.C.P., Assistant Medical Officer, Gordon Road Workhouse of the Camberwell Parish. **J. G. Emanuel**, M.D., B.S, B.Sc.Lond., M.B., Ch.B.Birm., M.R.C.P.Lond., Physician to Out-patients at the Birmingham and Midland Free Hospital for Sick Children. **R. H. Fagge**, M.R.C.S., L.R.C.P., District Medical Officer of the Melton Mowbray Union. **R. H. O. Garbutt**, L.R.C.P.&S.Edin., District Medical Officer of the Weardale Union. **L. M. T. Hungerford**, M.R.C.S., District Medical Officer at Perth, West Australia. **A. J. Hood**, M.B., Ch.M.Glasg., Visiting Medical Officer to the New South Wales Lunatic Asylum. **C. P. Johns**, M.R.C.S., L.R.C.P., Senior Medical Officer of the St. Pancras Parish Workhouse. **Alice Vowe Johnson**, F.R.C.S.I., D.P.H.Camb., Medical Officer to the Lambeth Poor-law Schools. **Charles Leedham-Green**, M.B., F.R.C.S., Honorary Surgeon to the Birmingham Children's Hospital. **H. C. McDouall**, M.R.C.S.Eng., L.R.C.P.Lond., Acting Medical Superintendent at the Hospital for Insane, Collan Park, Sydney. **G. E. Mackenzie**, M.B.Toronto, Clinical Assistant to the Chelsea Hospital for Women. **J. G. Macmillan**, M.B., Ch.M.Edin., Health Officer to the Kalgoorlie and Boulder District Board of Health. **R. F. Mudie**, M.B., C.M.Edin., Certifying Factory Surgeon for the Ladybank District, County Fife. **R. H. Norman**, M.D.Lond., B.S., M.R.C.S., L.R.C.P., Assistant Anæsthetist, Great Northern Central Hospital. **D. Price**, M.B.Lond., Certifying Factory Surgeon for the Works of Messrs. Boyd and Co., Castle Cary, county Somerset. **L. E. Stamm**, M.D., B.A., B.Sc., Medical Officer to the British Home and Hospital for Incurables, Streatham Common, S.W. **W. F. Thompson**, M.D.Edin., Medical Officer and Public Vaccinator for the No. District of the Launceston Union. **Wynn Martyn Westcott**, M.R.C.S.Eng., L.R.C.P.Lond., Resident Assistant Medical Officer, Heigham Hall Asylum, Norwich. **E. A. Woodward**, M.B., C.M.Edin., Government Medical Officer and Vaccinator at Wyalong, New South Wales. **H. C. Taylor Young**, C.M. M.D.Glasg., Honorary Assistant Gynæcological Surgeon to the Royal Prince Alfred Hospital, Sydney, New South Wales.

### "The Hospital" Institutional Library.

"Hospitals and Asylums of the World." 4 Vols., with a Portfolio of Plans, complete £12 12s.

"Burdett's Hospitals and Charities, 1903." 5s. net.

"Burdett's Official Nursing Directory, 1903." 8s. net.

"Cottage Hospitals, General, Fever, and Convalescent." 10s. 6d.

"Uniform System of Accounts for Hospitals and Public Institutions." New Edition. 4s. net.

"Hospital Expenditure: The Commissariat." 2s. 6d.

"A Legal Handbook for the Use of Hospital Authorities." 2s. 6d.

"The Cottage Hospital Case Book or Register of Patients." 10s. and 12s. 6d.

All these are published by the Scientific Press, Ltd., and may be obtained through any bookseller, or direct from the publishers, 28 and 29 Southampton Street, Strand, London, W.C.

**THE HANDBOOK OF THE OPEN-AIR TREATMENT**

By Dr. CHARLES REINHARDT

(Visiting Physician to Hailey Sanatorium, Wallingford, and Stourfield Park Sanatorium, Bournemouth),

Contains Views, Descriptions, and Information respecting 30 Sanatoria in Great Britain.

Price: Cloth, 2/6; Paper, 1/- (postage 4d.)

The "HEALTH RESORT" Publishing Office, 379 Strand, London, W.C.

**MEDICAL HISTORY FROM THE EARLIEST TIMES.**

By E. T. WITHINGTON, M.A., M.B. Oxon.

Demy 8vo., over 400 pp., with two Plates, cloth gilt, **12/6 net.**

"One of the best attempts that has yet been made in the English language to present the reader with a concise epitome of the history of medicine, and as such we very cordially commend it."

*Glasgow Medical Journal.*

THE SCIENTIFIC PRESS, LTD.,
28 and 29 Southampton Street, Strand, London, W.C.

### Special Text-Books for Nurses.

### OPHTHALMIC NURSING.

By SYDNEY STEPHENSON, F.R.C.S.E.

3/6 net, 3/10 post free.

A valuable guide on the Nursing of Eye Cases.

### NURSING IN DISEASES OF THE THROAT, NOSE, AND EAR.

By P. MACLEOD YEARSLEY, F.R.C.S.

2/6 post free.

Contains useful hints on these special subjects.

### THE ART OF MASSAGE.

By A. CREIGHTON HALE.

6/- post free.

Every Nurse must find a knowledge of Massage useful and interesting.

### FEVERS AND INFECTIOUS DISEASES.

By Dr. WILLIAM HARDING.

1/- post free.

An excellent little handbook.

### NOTES ON PHARMACY AND DISPENSING.

By C. J. S. THOMPSON.

1/- post free.

An admirable introduction to Dispensing.

### MENTAL NURSING.

By Dr. WILLIAM HARDING.

1/- post free.

Full of useful advice to Nurses in Mental Cases.

### OUTLINES OF INSANITY.

By Dr. WALMSLEY.

Paper covers 1/6 post free.

A Treatise on the Salient Features of Insanity. Very useful to the intelligent Mental Nurse.

*Obtainable of all Booksellers, and published by*
THE SCIENTIFIC PRESS, LIMITED, 28 & 29 Southampton Street, Strand, London,
*who will send their latest Catalogue of Nursing Text-books post free to any Nurse on application.*

### WORKS FOR NURSES.

**LESSONS ON MASSAGE.** By Mrs. MARGARET D. PALMER, Manager of the Massage Department and Instructor of Massage to the Nursing Staff of the London Hospital. Second Edition enlarged and revised; pp. xvi. + 262 with 118 Illustrations, plain and coloured. *Just Published.* Price 7/6 net.

**A MANUAL FOR STUDENTS OF MASSAGE.** By Miss M. A. ELLISON, L.O.S. Second Edition, edited by Miss G. MANLEY, pp. xii + 126. With 50 original Illustrations. *Just Published.* Price 3/6 net.

**A MANUAL FOR HOME NURSING.** By BERNARD MYERS, M.D., M.R.C.S., Surgeon and Lecturer to St. John's Ambulance Association. *Just Published.* Price 2/6 net.

**NOTES ON HOME NURSING.** By Miss D. M. GOLDIE, Lecturer on Nursing, Hygiene, &c., to the County Councils of London, Surrey and Middlesex. *Just Published.* Price 1/6 net.

**THE FEEDING AND HYGIENE OF INFANTS AND CHILDREN.** By JOHN McCAW, M.D., M.R.C.P., Physician to the Belfast Hospital for Children. *Just Published.* Price 2/6.

**DISEASES OF THE HAIR, INCLUDING GREYNESS, BALDNESS, &c., AND THEIR TREATMENT.** By DAVID WALSH, M.D., Western Hospital for Diseases of the Skin. Price 2/6 net.

**MENSTRUATION AND ITS DISORDERS.** By ARTHUR E. GILES, M.D., B.Sc., F.R.C.S., Surgeon Chelsea Hospital for Women. Price 2/6 net.

**MOVABLE ATLAS OF THE ANATOMY AND PHYSIOLOGY OF THE FEMALE GENERATIVE ORGANS AND OF PREGNANCY.** Second Edition. With Text. By ARTHUR E. GILES, M.D., F.R.C.S. Price 3/- net.

**MOVABLE ATLAS OF THE ANATOMY AND PHYSIOLOGY OF THE CHILD.** With Explanatory Text. By D'ARCY POWER, M.A.Oxon, M.B., F.R.C.S. Price 3/- net.

**BAILLIÈRE'S POPULAR MOVABLE ATLAS OF THE ANATOMY AND PHYSIOLOGY OF MAN.** Size 16 by 7½ inches. With Explanatory Text. By W. S. FURNEAUX, Special Science Teacher London School Board. Price 3/6 net.

London: BAILLIÈRE, TINDALL & COX, 8 Henrietta Street, Covent Garden.

**An admirable Food of the**

### EPPS'S

**Finest quality and flavour.**

### COCOA

**Nutritious and Economical.**

**LADIES AVOID CHILLS**

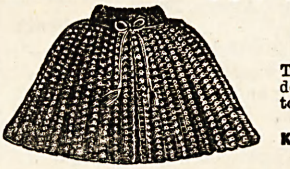

By wearing our Charming **KNITTED WOOL CAPES,** from **2/9** each.

They do not slip off like Shawls, nor do they hinder the movements. Write to-day for Illustrated List. Mention THE HOSPITAL.

**KNITTED CORSET & CLOTHING CO.,** 118 Mansfield Road, Nottingham.

***Just Published.***

### THE EXAMINATION OF THE URINE.

By J. K. WATSON, M.D.Edin., M.B., C.M.,

*Author of "A Handbook for Nurses."*

Price 1/- net; post free 1/1.

THE SCIENTIFIC PRESS, LIMITED, 28 & 29 Southampton Street, Strand, London, W.C.

Crown 8vo. paper cover, 6d.

**On Preparation for Operation in Private Houses.**

By STANMORE BISHOP, F.R.C.S.

**THE SCIENTIFIC PRESS, LTD., 28 & 29 Southampton Street, Strand, London, W.C.**

